# Accumulation or adaptation? A longitudinal study of role conflict and turnover intention among Chinese social workers

**DOI:** 10.1371/journal.pone.0359307

**Published:** 2026-10-23

**Authors:** Junhan Ji

**Affiliations:** 1 Yiwu Industrial & Commercial College, Yiwu, China; 2 Center for Social Work with Chinese Characteristics, YWICC, Yiwu, China; Middle Tennessee State University Jennings A Jones College of Business, UNITED STATES OF AMERICA

## Abstract

The Job Demands–Resources (JD-R) model suggests that job demands and resources influence employees’ turnover intention (TI), yet less is known about how these effects unfold over time. Drawing on the competing perspectives of accumulation and adaptation, this study examines whether TI among social workers is shaped by the cumulative effects of past role conflict (RC) or by responses to changing work conditions. Using matched panel data from the 2019 and 2022 waves of the China Social Work Longitudinal Study (CSWLS), the study investigates both lagged and change effects of RC and workplace support (WS). The results show that the association between baseline RC and subsequent TI becomes non-significant after controlling for prior TI. In contrast, changes in RC are positively associated with changes in TI, while changes in WS are negatively associated with changes in TI. Taken together, these findings indicate that baseline RC does not remain independently associated with subsequent TI after prior TI is taken into account, whereas changes in work conditions correspond to changes in TI over time. This study extends the JD-R model by introducing a longitudinal perspective and highlights the importance of considering changes in job demands and resources alongside their prior levels when examining turnover intention over time. Overall, the pattern of findings is more consistent with the adaptation perspective than with a persistent independent association of baseline RC with subsequent TI, contributing to a more dynamic understanding of workforce stability among social workers.

## Introduction

Workforce turnover among social workers has emerged as a critical issue of global concern [1,2]. Existing studies indicate that the social work sector is characterized by relatively high turnover rates. In some countries and regions, the annual turnover rate of social workers exceeds 20%–30% [3,4]. Such high turnover not only affects organizational efficiency but also poses significant challenges to service continuity and professional development. In this context, understanding the mechanisms underlying workforce mobility has become a central concern in the literature. Prior studies have consistently identified turnover intention (TI) as a key antecedent of actual turnover behavior and a strong predictor of employee mobility [2,5–8]. Therefore, examining the formation mechanisms of TI among social workers is critical for understanding workforce turnover in this field.

In order to explain why social workers develop TI, existing research has increasingly focused on role conflict (RC) in the work context. RC refers to a situation in which individuals face incompatible or inconsistent expectations from multiple role senders, making it difficult to satisfy these competing demands simultaneously [9]. In the field of social work, RC is particularly pronounced. Social workers are typically required to navigate multiple roles, including administrative, professional, and client-oriented roles. On the one hand, as organizational members, they are expected to complete various administrative and governance-related tasks in accordance with institutional arrangements. On the other hand, as professionals, they are obligated to deliver services consistent with professional ethics and practice standards. At the same time, in direct service provision, they must respond to the individualized needs of clients [10]. These roles often differ in their objectives and underlying logics. For instance, administrative tasks emphasize efficiency and compliance, and professional practice prioritizes ethical values and methodological rigor, while clients focus on the fulfillment of their individual needs. Such inconsistencies among role expectations increase the likelihood that social workers will experience RC, thereby elevating their TI [11–13]. Against this backdrop, examining TI among social workers from the perspective of RC offers a more contextually grounded explanation.

In recent years, the Job Demands–Resources (JD-R) model has been widely applied to explain the relationship between RC and TI [14,15]. Within this framework, RC is conceptualized as a typical job demand, requiring sustained effort to manage conflicting expectations from multiple role sources, which in turn leads to psychological strain and resource depletion. In contrast, workplace support (WS), including support from supervisors and colleagues, is regarded as a key job resource that provides emotional support, informational, and instrumental assistance, thereby enhancing individuals’ capacity to cope with work-related stress [16,17].

According to the JD-R model, job demands and job resources influence work outcomes through different pathways. On the one hand, high levels of job demands (e.g., RC) contribute to strain accumulation through a “health impairment process,” thereby increasing TI. On the other hand, job resources (e.g., WS) operate through a “motivational process,” alleviating stress, sustaining work engagement, and ultimately reducing TI [14,18,19]. In the field of social work, prior studies have examined these relationships from relevant theoretical perspectives. Regarding the health impairment process, Ji found that stressors such as RC significantly increase TI among social workers [20]. Similarly, Kim and Stoner [4], based on a sample of social workers, reported that work stress, particularly that arising from multiple and competing role demands, is positively associated with TI [4]. With respect to the motivational process, Schaufeli and Bakker [21] demonstrated that job resources, such as WS, can mitigate the negative effects of RC on TI within the JD-R framework [21].

Existing studies have consistently demonstrated that role conflict is positively associated with turnover intention among social workers. However, most of this evidence is based on cross-sectional data and therefore provides limited insight into how the effects of role conflict unfold over time. Although previous studies have established that role conflict matters, it remains unclear whether turnover intention is primarily driven by past experiences of role conflict or by changes in role conflict over time.

One possible explanation is the accumulation perspective. This perspective suggests that repeated exposure to role conflict gradually depletes employees’ psychological resources and increases their intention to leave [20]. Under this view, social workers who experienced high levels of role conflict in the past should continue to report higher turnover intention in the future. Even after several years, the negative effects of earlier role conflict are expected to persist [22].

An alternative explanation is the adaptation perspective. Employees may gradually adapt to chronically high levels of role conflict and develop new expectations about their work environment [23]. If adaptation occurs, the influence of past role conflict should weaken over time. In this case, turnover intention may be shaped less by historical levels of role conflict and more by whether role conflict is increasing or decreasing. Similarly, changes in workplace support may play a critical role in influencing changes in turnover intention.

These two perspectives imply different expectations regarding the relationship between role conflict and turnover intention. The accumulation perspective predicts that baseline role conflict should continue to predict future turnover intention, whereas the adaptation perspective predicts that changes in role conflict and workplace support should be more strongly associated with changes in turnover intention. Despite the importance of this distinction, few longitudinal studies have directly examined these competing explanations among social workers.

To address these limitations, the present study re-examines the relationship between RC and TI from a temporal perspective. Although previous research has consistently documented a positive association between RC and TI, it remains unclear whether this relationship reflects the enduring effects of past role conflict or employees’ adaptive responses to changing work conditions over time. Accordingly, this study seeks to empirically evaluate the competing explanations offered by the accumulation perspective and the adaptation perspective.

Specifically, the analysis is conducted at two levels. First, the study examines the effect of baseline RC measured in 2019 on subsequent TI measured in 2022. This analysis assesses whether role conflict exerts a lasting influence on turnover intention over time. If the accumulation perspective is supported, prior RC should continue to predict future TI even after a substantial time interval. Second, the study investigates the relationship between changes in RC and changes in TI over the same period. In addition, changes in WS are incorporated into the analysis to examine whether evolving job demands and job resources are more influential than historical levels in shaping turnover intention. If the adaptation perspective holds, changes in RC and WS should be more strongly associated with changes in TI than baseline RC.

To implement this design, the study draws on matched panel data from the China Social Work Longitudinal Study (CSWLS) collected in 2019 and 2022. Baseline RC measured in 2019 is used to assess the long-term effect of job demands on subsequent turnover intention, while change variables are constructed by calculating differences between the two waves. This approach enables an examination of both the enduring and dynamic effects of work experiences on turnover intention.

This study makes two primary contributions to the literature. First, it extends the application of the JD-R model in turnover research by introducing a longitudinal perspective. While existing studies have primarily focused on the static relationship between RC and TI, the present study examines how baseline RC and changes in working conditions are associated with TI over time. In doing so, it provides longitudinal evidence regarding whether TI is more closely associated with the cumulative effects of past RC or with adaptive responses to changes in job demands and job resources.

Second, this study advances the conceptualization of RC as a job demand. Rather than focusing solely on its absolute level, the study highlights the importance of changes in RC and WS over time. By emphasizing longitudinal changes in work experiences, the findings contribute to a more nuanced understanding of how social workers’ career decisions are associated with changing work conditions.

The remainder of this paper is organized as follows. The next section develops the theoretical framework and research hypotheses, followed by the Methods and Results sections. The Discussion section interprets the findings and their implications, and the final section presents the conclusions and limitations.

## Theoretical framework and hypotheses

### The JD-R model

The JD-R model is a widely recognized theoretical framework for explaining the relationship between work contexts and work outcomes. Originally proposed by Demerouti et al. [14] and further developed by Bakker and Demerouti [18], the model integrates the effects of diverse job characteristics on individuals’ behavioral and psychological outcomes [18,24]. Within this framework, work-related factors are categorized into two broad types: job demands and job resources. Job demands refer to aspects of the job that require sustained effort and are therefore associated with physiological and psychological costs, whereas job resources refer to factors that help individuals cope with job demands, achieve work goals, and facilitate personal growth and development [21].

Based on this classification, the JD-R model proposes two fundamental processes. On the one hand, job demands affect individuals through a health impairment process, whereby prolonged exposure to high demands leads to the accumulation of strain and adverse outcomes. On the other hand, job resources operate through a motivational process, enhancing individuals’ capacity to adapt to their work and buffering the negative effects of job demands. Together, these processes jointly constitute the core mechanism through which work environments shape individual behavior.

It should be noted that the JD-R model covers a wide range of variables, and empirical studies typically select specific constructs based on their research objectives. Consistent with the aims of this study, RC is conceptualized as a representative job demand, WS is treated as a key job resource, and TI is specified as the outcome variable. This specification enables a focused examination of the relationship between RC and TI while maintaining alignment with the core logic of the JD-R model and ensuring a parsimonious analytical framework.

### Accumulation and adaptation perspectives on TI

Although prior research consistently reports a positive relationship between RC and TI, less attention has been paid to how this relationship unfolds over time. Two competing perspectives provide different expectations.

The accumulation perspective suggests that repeated exposure to RC gradually depletes employees’ psychological resources and generates enduring negative consequences [15]. Under this view, RC experienced at an earlier stage should continue to influence TI in later periods. Accordingly, social workers who experience high levels of role conflict may remain vulnerable to TI even after a considerable period of time [9,22,25].

In contrast, the adaptation perspective argues that employees may gradually adjust to chronic job demands and establish a new psychological equilibrium. If adaptation occurs, the effects of past role conflict should weaken over time. Instead, employees may become more sensitive to changes in work conditions [18]. Under this view, increases in RC and decreases in WS are expected to exert stronger effects on TI than historical levels of these variables [23].

These competing perspectives raise an important question: Is TI primarily driven by the cumulative effects of past RC or by adaptive responses to changing work conditions? Drawing on the accumulation perspective, this study first examines whether baseline RC exerts a lasting influence on subsequent TI.

Hypothesis 1 (H1): $RC_{\text{2}\text{0}\text{1}\text{9}}$ is positively associated with $TI_{\text{2}\text{0}\text{2}\text{2}}$.

### Dynamic changes in RC and TI

Within the JD-R framework, RC, as a typical job demand, plays a critical role in shaping TI [16,20]. Building on this perspective, emerging research suggests that individuals evaluate their work conditions not only based on the current level of job demands but also on how these demands evolve over time [3,23,26]. In other words, changes in work experiences may exert a stronger influence on individuals’ judgments and behavioral decisions than their static levels.

From this perspective, variations in RC are likely to have a more direct impact on individuals’ decisions to remain in or leave their positions. When RC increases over time, individuals may perceive that conflicts among role demands are intensifying. This heightened and persistent experience of conflict repeatedly exposes individuals to situations in which they are unable to satisfy multiple expectations simultaneously, thereby reinforcing the perception that the job is difficult to sustain. Prior research suggests that employees are sensitive to changes in work experiences and continuously re-evaluate their work environment over time [23]. Under such conditions, individuals are more likely to view leaving their current positions as a coping strategy, thereby increasing their TI.

Conversely, when RC decreases over time, individuals encounter fewer conflicts among role demands, and the difficulty of managing their work is reduced. This improvement may lead individuals to perceive their work as more manageable, thereby lowering their motivation to leave. Thus, changes in RC not only reflect shifts in job demands but also directly shape individuals’ evaluations of work sustainability.

Accordingly, changes in RC are translated into changes in TI through individuals’ ongoing evaluations of their work conditions. Based on the above analysis, the following hypothesis is proposed:

Hypothesis 2 (H2): Changes in RC are positively associated with changes in TI.

### Dynamic changes in WS and TI

Although this study primarily focuses on the dynamic effects of RC, WS is incorporated to provide a more comprehensive understanding of the formation of TI.

Within the JD-R framework, WS is regarded as a key job resource that enables individuals to cope with job demands effectively [21,27]. Unlike job demands, job resources facilitate the management of complex tasks by providing various forms of support, thereby reducing the perceived difficulty of work.

In the context of social work, WS typically originates from organizations, supervisors, and colleagues. When individuals perceive a high level of WS, they are more likely to receive assistance in dealing with multiple role demands, which enhances their coping capacity. Under such conditions, even in the presence of RC, individuals are more likely to perceive their work as manageable and therefore less inclined to leave their positions.

In contrast, when WS is limited, individuals must address work-related challenges largely on their own, making it more difficult to sustain their work. Under such circumstances, leaving the current position may be perceived as a more viable option, thereby increasing TI. Previous studies consistently report a negative association between WS and TI [3,17,28]. Changes in workplace support may influence employees’ evaluations of their work environment and their willingness to remain in their current positions. An increase in WS may enhance employees’ coping capacity and reduce TI, whereas a decline in WS may weaken employees’ ability to manage job demands and increase TI. Accordingly, changes in WS are expected to be negatively associated with changes in TI. Based on the above analysis, the following hypothesis is proposed:

Hypothesis 3 (H3): Changes in WS are negatively associated with changes in TI.

## Methods

### Research design

This study employs a longitudinal quantitative research design using matched panel data from the 2019 and 2022 waves of the CSWLS. The two-wave panel structure enables us to distinguish between associations involving prior levels of work conditions and those involving within-person changes over time.

Within the JD-R framework, the study focuses on RC as a job demand and WS as a job resource in relation to TI. To examine the temporal patterns underlying these relationships, two complementary analytical approaches are used. First, a lagged outcome model examines whether RC measured in 2019 is associated with TI measured in 2022. Baseline TI is subsequently included in the model to account for the temporal stability of turnover intention and to assess whether baseline RC is associated with subsequent TI beyond respondents’ pre-existing turnover intentions. Second, a change-score approach examines whether within-person changes in RC between 2019 and 2022 are associated with corresponding changes in TI. Changes in WS are also included in the change-score model to examine whether changes in this job resource are associated with changes in TI over the same period.

The use of both approaches is intended to address different aspects of the longitudinal relationship. The lagged outcome model evaluates whether earlier RC has an association with later TI after accounting for baseline TI, whereas the change-score model focuses on whether changes in work conditions correspond to changes in TI within individuals. Because each approach relies on different analytical assumptions, the findings are further evaluated using an alternative baseline-adjusted specification that simultaneously includes RC and WS measured at both waves together with baseline TI. This additional specification assesses whether the main findings are robust to an alternative operationalization of longitudinal change.

Multiple regression analysis is used to estimate all models. Consistent with previous research on social workers’ TI, gender, age, marital status, education level, party membership, and income are included as covariates [1,3,17,19]. All statistical analyses are conducted using SPSS Statistics Version 22.0.

### Data source and data processing

The data used in this study are drawn from the CSWLS, a nationwide survey conducted by the School of Social and Public Administration at East China University of Science and Technology. Launched in 2019 and administered every three years, the CSWLS is designed to systematically collect information on the development of the social work sector and the professional conditions of social workers in China.

In terms of sampling design, the CSWLS adopts a stratified approach based on the number of social work institutions and city size. Cities are classified into two groups: (1) large cities with a relatively high number of social work institutions (e.g., municipalities and provincial capitals), and (2) smaller cities with fewer institutions. Based on this classification, samples are allocated proportionally across strata, and a multi-stage random sampling method is employed to select respondents.

Specifically, in the first stage, social work institutions are randomly selected within each stratum according to a predetermined sampling quota. In the second stage, social workers are selected from each sampled institution using systematic sampling based on staff rosters. When the size of an institution is insufficient, all social workers within that institution are included. In cases where selected institutions are unable to participate or access to respondents is limited, replacement institutions are drawn from the same city-level sampling frame according to predefined procedures to preserve the overall sample structure. In addition, to facilitate follow-up surveys, some large institutions not initially selected may be incorporated into the sampling frame.

To construct the longitudinal panel, the 2022 wave prioritized follow-up interviews with respondents who had participated in the 2019 survey, using previously collected contact information. For individuals who had left the profession or could not be reached, new respondents were recruited as part of the 2022 cross-sectional sample. These newly recruited respondents were not included in the longitudinal analyses because they did not have corresponding baseline observations.

Using unique respondent identification codes, respondents were matched across the two survey waves. The 2019 baseline survey yielded 5,965 valid responses, while the 2022 survey yielded 6,499 valid responses. The matching procedure identified 1,744 respondents who participated in both waves, constituting the matched longitudinal panel from which model-specific analytical samples were derived. Because the availability of complete observations varied across study variables, the effective sample size differed across statistical models. The regression analyses therefore report model-specific sample sizes rather than treating all analyses as having a single uniform analytical sample. The sample construction process is summarized in S1 Table in S1 File.

With respect to data processing, consistency checks were conducted to ensure comparability of variables across the two waves. In the raw dataset, variables contained both user-defined missing-value codes (e.g., −9999) and system missing values. User-defined missing-value codes were treated as missing before analysis. For multi-item measures, composite scores were calculated from the available item responses according to the scoring procedures used in constructing the study variables.

Missing data in the regression analyses were handled using listwise deletion in SPSS; thus, each model included only respondents with valid observations for all variables entered in that particular model. Consequently, analytical sample sizes varied according to the variables included in each specification. For example, the fully adjusted lagged model included 1,624 respondents, whereas the fully adjusted change-score model included 1,612 respondents. No missing values were imputed. Model-specific sample sizes are reported in the corresponding results tables to make this procedure transparent [29].

Given the attrition between the two survey waves, an attrition analysis was conducted to compare respondents retained in the matched panel with those who were not successfully followed up. Independent-samples t-tests indicated no significant differences in baseline RC or WS between the two groups. Respondents retained in the panel were, however, slightly older and reported somewhat lower TI at baseline. Although selective attrition cannot be completely ruled out, the small magnitude of these differences and the absence of significant differences in baseline RC and WS suggest that attrition did not substantially alter the panel with respect to the focal work-condition variables. Detailed attrition results are provided in S4 Table in S1 File.

This study used anonymized secondary data from the 2019 and 2022 waves of the CSWLS. The original CSWLS data collection was conducted by the School of Social and Public Administration at East China University of Science and Technology in accordance with the principles of the Declaration of Helsinki and was approved by the Ethics Committee of East China University of Science and Technology. Written informed consent was obtained from participants as part of the original data collection, and the authors had no access to personally identifiable information. The data are subject to third-party restrictions imposed by the CSWLS research team and therefore cannot be publicly shared by the authors. Qualified researchers may request access to the original data directly from the School of Social and Public Administration at East China University of Science and Technology.

### Measurement

#### RC.

RC was measured using items adapted from the role conflict scale [30]. To ensure comparability across the two survey waves, the longitudinal analyses used the eight RC items that were administered with equivalent wording in both 2019 and 2022. The items were rated on a five-point Likert scale ranging from 1 (strongly disagree) to 5 (strongly agree), with higher mean scores indicating greater levels of RC. Although the original scale was developed in Western organizational settings, previous studies have successfully applied it among Chinese social workers and demonstrated satisfactory psychometric properties [20]. The eight-item measure demonstrated good internal consistency in both waves, with Cronbach’s alpha values of 0.799 in 2019 and 0.831 in 2022.

#### TI.

TI was measured using a three-item intention-to-leave scale [31]. The items include “I plan to leave my current institution within the next six months,” “I may leave my current institution within the next three years,” and “I occasionally have the idea of leaving my current organization.” Responses were recorded on a five-point Likert-type scale ranging from 1 (strongly disagree) to 5 (strongly agree), with higher scores indicating stronger TI. The same three-item measure was administered in both the 2019 and 2022 waves. The scale has been widely used in studies of human service professionals and Chinese social workers, demonstrating good reliability and construct validity [1,28]. The scale exhibited acceptable internal consistency in both waves, with Cronbach’s alpha values of 0.788 in 2019 and 0.790 in 2022.

#### WS.

WS was assessed using the Workplace Support Scale, adapted to capture support within the organizational context [32]. The measure comprises 24 items covering six aspects of workplace support across four organizational sources: direct supervisors, immediate supervisors, peer colleagues, and senior organizational leaders. The six aspects assess whether these organizational members provide reliable support when difficulties arise, listen to work-related problems, assist with work tasks, demonstrate adequate work competence, effectively handle their own work responsibilities, and recognize employees’ good performance. Each item was rated on a five-point scale ranging from 1 (“very dissatisfied”) to 5 (“very satisfied”), and item scores were averaged to represent the overall level of perceived WS, with higher scores indicating greater perceived support.

Following the CSWLS design, the measure reflects the organizational structure of Chinese social work agencies and has been used in previous studies of Chinese social workers [8,31,32]. The same 24 items were used to construct WS in both the 2019 and 2022 waves, thereby ensuring consistency in measurement across waves. The measure demonstrated excellent internal consistency in both waves, with Cronbach’s alpha values of 0.955 in 2019 and 0.960 in 2022. The measurement characteristics of the focal variables are summarized in S2 Table in S1 File, while the operational definitions and coding of the study variables are provided in S6 Table in S1 File.

### Data analysis

All statistical analyses were conducted using SPSS Version 22.0. Descriptive statistics and Pearson correlation analyses were first performed to summarize the study variables and examine their bivariate relationships. For the focal longitudinal variables, descriptive statistics at each wave were calculated among respondents with valid observations for the corresponding variable in both waves, thereby ensuring that the 2019 and 2022 estimates for each construct were based on the same respondents.

Given the longitudinal design of the study, CMB is less likely to fully account for the observed relationships because the focal variables were assessed across two survey waves. Nevertheless, Harman’s single-factor test was conducted as an additional diagnostic procedure. All measurement items were entered into an exploratory factor analysis, and the proportion of variance explained by the first unrotated factor was examined.

To address the research question, two complementary longitudinal analytical approaches were used. First, lagged regression models examined whether baseline RC measured in 2019 was associated with TI measured in 2022. The models were estimated hierarchically, with demographic covariates included before baseline TI was added to the final specification. Controlling for TI in 2019 allowed us to examine whether baseline RC was associated with subsequent TI beyond the temporal stability of TI itself.

Second, change-score models examined whether within-person changes in RC between 2019 and 2022 were associated with corresponding changes in TI. Change scores were calculated as the 2022 value minus the corresponding 2019 value. The extended change-score model additionally included change in WS to examine whether changes in this job resource were associated with changes in TI after accounting for changes in RC and demographic covariates. The lagged and change-score approaches address complementary aspects of the longitudinal relationship: the former evaluates the prospective association between an earlier exposure and a later outcome after adjustment for the baseline outcome, whereas the latter evaluates whether within-person changes in work conditions correspond to within-person changes in TI. The construction of change variables is detailed in S3 Table in S1 File.

The primary regression models were specified as follows:

(1) Lagged model

$$\begin{array}{c} {TI}_{\text{2022}}=\beta_{\text{0}}+\beta_{\text{1}}\cdot {RC}_{\text{2019}}+\beta_{\text{2}}\cdot {TI}_{\text{2019}}+\beta_{\text{3}}\cdot \text{Controls}+\varepsilon \end{array}$$

(2) Change model

$$\begin{array}{c} \Delta TI=\beta_{o}+\beta_{\text{1}}\cdot \Delta RC+\beta_{\text{2}}\cdot \text{Controls}+\varepsilon \end{array}$$

(3) Extended change model

$$\begin{array}{c} \Delta TI=\beta_{o}+\beta_{\text{1}}\cdot \Delta RC+\beta_{\text{2}}\cdot \Delta WS+\beta_{\text{3}}\cdot \text{Controls}+\varepsilon \end{array}$$

where TI denotes turnover intention, RC denotes role conflict, WS denotes workplace support, and Δ denotes the difference between the 2022 and 2019 scores. The covariates included gender, age, marital status, education level, party membership, and income.

To assess whether the substantive findings were sensitive to the choice of longitudinal specification, an additional baseline-adjusted model was estimated in which RC and WS measured in both 2019 and 2022 were entered simultaneously, together with baseline TI and the demographic covariates. This alternative specification evaluates whether the associations of the 2022 work conditions with TI in 2022 remain evident after accounting for their corresponding baseline levels and baseline TI. Because RC, WS, and TI measured in 2022 were assessed contemporaneously, coefficients involving the 2022 measures are interpreted as conditional associations rather than prospective or causal effects.

Missing observations in the regression analyses were handled using listwise deletion. Consequently, the effective analytical sample varied across model specifications according to the availability of complete observations for the variables included in each model, and model-specific sample sizes are reported in the corresponding tables.

Several diagnostic and supplementary analyses were conducted to evaluate the robustness of the results. Given the attrition between survey waves, an attrition analysis compared respondents retained in the matched panel with those who were not successfully followed up. Multicollinearity was assessed using variance inflation factors (VIFs). For the fully adjusted change-score model, examination of the residual distribution and influence statistics indicated no marked departure from residual normality or highly influential observations; VIFs ranged from 1.018 to 1.486, and the maximum Cook’s distance was 0.019. Detailed information on sample construction, variable construction, change-score computation, attrition analysis, and supplementary analyses is provided in S1 File. The data processing and statistical analysis procedures are summarized in S7 Table.

### Attrition analysis

To assess potential attrition bias, baseline characteristics were compared between respondents retained in the longitudinal sample and those lost to follow-up. Table 1 indicated no significant differences in baseline role conflict (p = 0.611) or workplace support (p = 0.285) between the two groups. Retained respondents were slightly older (p = 0.002) and reported somewhat lower turnover intention at baseline (p < 0.001). However, effect sizes were small (Cohen’s d = 0.085 and 0.197, respectively), suggesting limited practical differences between retained and attrited respondents. Overall, the findings indicate that attrition bias is unlikely to substantially affect the substantive conclusions of this study.

**Table 1:** Attrition analysis.

Variable	Attrited respondents	Retained respondents	p-value	Cohen’s d
**Age**	33.22	33.90	0.002	0.085
${RC}_{2019}$	2.75	2.76	0.611	0.015
${TI}_{2019}$	2.61	2.43	<0.001	0.197
${WS}_{2019}$	4.10	4.08	0.285	0.031

## Results

### Assessment of CMB and multicollinearity

Because the study used a longitudinal design in which the focal constructs were assessed across two survey waves, temporal separation may reduce, although not eliminate, concerns regarding common method bias (CMB). Harman’s single-factor test was therefore conducted as an additional diagnostic procedure. The first unrotated factor accounted for 31.60% of the total variance, below the commonly used 40% threshold. Although this diagnostic cannot rule out CMB, the result provides no indication that a single common factor dominated the covariance among the measured variables.

Multicollinearity was assessed using variance inflation factors (VIFs) and tolerance values across the regression specifications. VIF values did not exceed 1.541, and all tolerance values were well above 0.10, indicating no evidence of problematic multicollinearity.

### Descriptive statistics

Table 2 presents the descriptive statistics of the sample and the focal study variables. The respondents had a mean age of 33.90 years (SD = 7.40), and the majority were female (79.6%). Slightly more than half were married (52.4%), and 26.2% were members of the Communist Party of China. The average level of education was 15.74 years (SD = 1.38). With respect to the focal variables, TI increased from 2.43 (SD = 0.88) in 2019 to 2.62 (SD = 0.88) in 2022, with a mean within-person change of 0.18 (SD = 1.02). RC also increased, from 2.76 (SD = 0.62) in 2019 to 2.92 (SD = 0.66) in 2022, with a mean change of 0.16 (SD = 0.75). In contrast, WS declined from 4.08 (SD = 0.75) in 2019 to 3.93 (SD = 0.80) in 2022, with a mean change of −0.15 (SD = 0.89). These descriptive patterns indicate modest increases in TI and RC and a modest decline in WS across the two survey waves.

**Table 2:** Descriptive statistics of the sample and focal variables.

Variables	Proportion (%) or mean (SD)
**Demographic characteristics**	
**Male**	20.4%
**Female**	79.6%
**Married**	52.4%
**Unmarried**	47.6%
**Communist party member**	26.2%
**Non-communist party member**	73.8%
**Age**	33.90 (7.40)
**Education**	15.74 (1.38)
**Income**	4.49 (2.39)
**Key variables**	
${RC}_{2019}$	2.76 (0.62)
${RC}_{2022}$	2.92 (0.66)
ΔRC	0.16 (0.75)
${TI}_{2019}$	2.43 (0.88)
${TI}_{2022}$	2.62 (0.88)
ΔTI	0.18 (1.02)
${WS}_{2019}$	4.08 (0.75)
${WS}_{2022}$	3.93 (0.80)
ΔWS	−0.15 (0.89)

**Note.** SD, standard deviation; RC, role conflict; TI, turnover intention; WS, workplace support. Descriptive statistics for each focal variable across the two waves were calculated among respondents with valid observations for that variable in both waves (TI: N = 1,744; RC: N = 1,743; WS: N = 1,729). Δ denotes the 2022 score minus the corresponding 2019 score.

### Correlation analysis

Table 3 presents the means, standard deviations, and correlations among the focal study variables within the matched longitudinal panel. RC in 2019 was positively correlated with TI in both 2019 (r = 0.288, p < 0.001) and 2022 (r = 0.133, p < 0.001). RC in 2022 was also positively correlated with TI in 2022 (r = 0.302, p < 0.001). In addition, changes in RC were positively correlated with changes in TI (r = 0.198, p < 0.001), whereas changes in WS were negatively correlated with changes in TI (r = −0.198, p < 0.001).

**Table 3:** Means, standard deviations, and correlations.

Variables	M	SD	1	2	3	4	5	6	7	8	9
${RC}_{2019}$	2.76	0.62	—								
${RC}_{2022}$	2.92	0.66	0.317^***^	—							
ΔRC	0.16	0.75	−0.552^***^	0.617^***^	—						
${TI}_{2019}$	2.43	0.88	0.288^***^	0.187^***^	−0.075^**^	—					
${TI}_{2022}$	2.62	0.88	0.133^***^	0.302^***^	0.155^***^	0.327^***^	—				
ΔTI	0.18	1.02	−0.134^***^	0.099^***^	0.198^***^	−0.582^***^	0.578^***^	—			
${WS}_{2019}$	4.08	0.75	−0.343^***^	−0.144^***^	0.157^***^	−0.251^***^	−0.118^***^	0.116^***^	—		
${WS}_{2022}$	3.93	0.80	−0.198^***^	−0.325^***^	−0.121^***^	−0.169^***^	−0.302^***^	−0.113^***^	0.345^***^	—	
ΔWS	−0.15	0.89	0.106^***^	−0.176^***^	−0.242^***^	0.060^*^	−0.170^***^	−0.198^***^	−0.533^***^	0.610^***^	—

Note. M, mean; SD, standard deviation; RC, role conflict; TI, turnover intention; WS, workplace support. Correlations were calculated within the matched longitudinal panel using pairwise available observations; sample sizes ranged from 1,729–1,744. Δ denotes the 2022 score minus the corresponding 2019 score. * p < 0.05, ** p < 0.01, *** p < 0.001.

More importantly, ΔRC was positively correlated with ΔTI (r = 0.198, p < 0.001), providing preliminary evidence that within-person changes in RC were associated with corresponding changes in TI over the study period. Baseline TI was negatively correlated with ΔTI (r = −0.582, p < 0.001), indicating that respondents with higher initial levels of TI tended to show smaller increases, or greater decreases, in TI between 2019 and 2022.

WS was negatively correlated with RC and TI at both measurement waves. In addition, ΔWS was negatively correlated with ΔTI (r = −0.198, p < 0.001), indicating that increases in WS were associated with decreases in TI. ΔWS was also negatively correlated with ΔRC (r = −0.242, p < 0.001), indicating that increases in WS tended to coincide with decreases in RC over the same period.

### Regression results

Table 4 presents the hierarchical regression results predicting ${TI}_{\text{2}\text{0}\text{2}\text{2}}$. As shown in Model 1, ${RC}_{\text{2}\text{0}\text{1}\text{9}}$ was positively and significantly associated with ${TI}_{\text{2}\text{0}\text{2}\text{2}}$ (B = 0.188, p < 0.001).

**Table 4:** Hierarchical regression results.

DV: ${TI}_{2022}$	Model 1	Model 2	Model 3
**Effect**	**B (SE)**	**B (SE)**	**B (SE)**
**Gender**		−0.037 (0.052)	0.002 (0.050)
**Age**		−0.024^***^ (0.003)	−0.017^***^ (0.003)
**Marital status**		−0.138^**^ (0.048)	−0.116^*^ (0.047)
**Party membership**		−0.049 (0.050)	−0.027 (0.048)
**Education**		−0.005 (0.016)	−0.004 (0.016)
**Income**		−0.022^*^ (0.009)	−0.018^*^ (0.009)
$RC_{2019}$	0.188^***^ (0.034)	0.144^***^ (0.034)	0.045 (0.034)
$TI_{2019}$		—	0.262^***^ (0.025)
**Observations (N)**	1,743	1,624	1,624
**F-statistics**	31.391^***^	22.962^***^	35.434^***^
**Adjusted R²**	0.017	0.087	0.145
**△R²**		0.073^***^	0.059^***^

Note. Unstandardized coefficients (B) are reported with standard errors in parentheses. * p < 0.05, ** p < 0.01, *** p < 0.001.

After including demographic control variables in Model 2, the effect of $RC_{\text{2}\text{0}\text{1}\text{9}}$ remained significant, although its magnitude decreased (B = 0.144, p < 0.001). Among the control variables, age, marital status, and income were negatively associated with $TI_{\text{2}\text{0}\text{2}\text{2}}$.

In Model 3, $TI_{\text{2}\text{0}\text{1}\text{9}}$ was added to the model. The results indicate that $TI_{\text{2}\text{0}\text{1}\text{9}}$ was a strong predictor of $TI_{\text{2}\text{0}\text{2}\text{2}}$ (B = 0.262, p < 0.001). However, after controlling for $TI_{\text{2}\text{0}\text{1}\text{9}}$, the effect of $RC_{\text{2}\text{0}\text{1}\text{9}}$ became non-significant (B = 0.045, p = 0.188).

These findings suggest that the observed relationship between RC and TI over time may largely reflect the stability of TI rather than an independent long-term effect of RC. In other words, $RC_{\text{2}\text{0}\text{1}\text{9}}$ did not significantly predict $TI_{\text{2}\text{0}\text{2}\text{2}}$ once prior levels of TI were taken into account.

Taken together, the results indicate that baseline RC has limited explanatory power for future TI beyond employees’ initial TI. This finding highlights the importance of considering dynamic changes in working conditions rather than relying solely on baseline measures when examining TI over time. Therefore, H1 was not supported.

### Change model results

Table 5 presents the regression results for changes in TI between 2019 and 2022. In Model 1, ΔRC was positively associated with ΔTI (B = 0.271, p < 0.001), indicating that increases in RC were associated with increases in TI over the study period. This association remained statistically significant after demographic covariates were included in Model 2 (B = 0.274, p < 0.001).

**Table 5:** Hierarchical regression results for change in turnover intention.

DV: ΔTI	Model 1	Model 2	Model 3
**Effect**	**B (SE)**	**B (SE)**	**B (SE)**
**Gender**		0.072 (0.062)	0.080 (0.062)
**Age**		0.006 (0.004)	0.004 (0.004)
**Marital status**		−0.038 (0.057)	−0.007 (0.057)
**Party membership**		0.043 (0.059)	0.031 (0.059)
**Education**		−0.001 (0.020)	−0.001 (0.019)
**Income**		−0.006 (0.011)	−0.005 (0.011)
**ΔRC**	0.271^***^ (0.032)	0.274^***^ (0.033)	0.221^***^ (0.034)
**ΔWS**			−0.175^***^ (0.029)
**Observations (N)**	1,743	1,624	1,612
**F-statistics**	71.349^***^	10.285^***^	13.546^***^
**R²**	0.039	0.043	0.063
**Adjusted R²**	0.039	0.039	0.059
**△R²**		0.004	0.020

Note. Unstandardized coefficients (B) are reported with standard errors in parentheses. * p < 0.05, ** p < 0.01, *** p < 0.001.

In Model 3, ΔWS was additionally included as a time-varying job resource. ΔWS was negatively associated with ΔTI (B = −0.175, p < 0.001), indicating that increases in WS were associated with decreases in TI. At the same time, ΔRC remained positively associated with ΔTI (B = 0.221, p < 0.001). Thus, changes in both job demands and job resources were independently associated with changes in TI after adjustment for the demographic covariates.

Taken together, the change-score results indicate that increases in RC were associated with increases in TI, whereas increases in WS were associated with decreases in TI. These findings are consistent with the adaptation perspective, according to which changes in current working conditions correspond to changes in employees’ turnover intentions. Accordingly, H2 and H3 were supported.

### Robustness checks

To assess whether the findings were sensitive to the choice of longitudinal specification, an alternative baseline-adjusted model was estimated in which RC and WS measured in both 2019 and 2022 were entered simultaneously, together with TI in 2019 and the demographic covariates. This specification provides an alternative to the change-score approach by examining whether work conditions measured in 2022 were associated with TI in 2022 after accounting for their corresponding baseline levels and baseline TI.

As shown in Table 6, RC in 2022 was positively associated with TI in 2022 (B = 0.234, p < 0.001), whereas WS in 2022 was negatively associated with TI in 2022 (B = −0.225, p < 0.001). In contrast, neither RC in 2019 (B = −0.058, p = 0.093) nor WS in 2019 (B = 0.018, p = 0.532) was independently associated with TI in 2022 in this specification. Baseline TI remained positively associated with TI in 2022 (B = 0.223, p < 0.001). The model explained 23.4% of the variance in TI in 2022 (R^2^ = 0.234; adjusted R^2^ = 0.228). The detailed robustness analysis results are also presented in S5 Table in S1 File.

**Table 6:** Robustness check using an alternative baseline-adjusted specification.

DV: $TI_{2022}$	Robustness model
**Gender**	−0.042 (0.048)
**Age**	−0.016^***^ (0.003)
**Marital status**	−0.077 (0.044)
**Party membership**	−0.028 (0.046)
**Education**	−0.012 (0.015)
**Income**	−0.021^*^ (0.009)
$RC_{2019}$	−0.058 (0.035)
$RC_{2022}$	0.234^***^ (0.032)
$𝐖S_{2019}$	0.018 (0.029)
$𝐖S_{2022}$	−0.225^***^ (0.027)
$TI_{2019}$	0.223^***^ (0.024)
**Observations (N)**	1,612
**F-statistics**	44.328^***^
**R²**	0.234
**Adjusted R²**	0.228

Note. Unstandardized coefficients (B) are reported with standard errors in parentheses. * p < 0.05, ** p < 0.01, *** p < 0.001.

These results are consistent with the pattern observed in the primary analyses. Specifically, the change-score models showed that changes in RC and WS were associated with changes in TI, while the alternative baseline-adjusted specification showed that RC and WS measured in 2022 remained associated with TI in 2022 after their corresponding baseline levels and baseline TI were taken into account. Because the 2022 measures were assessed contemporaneously, these coefficients should be interpreted as conditional associations rather than prospective or causal effects. Overall, the convergence of findings across the two specifications provides additional evidence that the observed pattern is more consistent with an adaptation perspective than with a persistent independent association of baseline work conditions with subsequent TI.

## Discussion

### Interpretation of the findings

Drawing on the JD-R framework and longitudinal data from the 2019 and 2022 waves of the CSWLS, this study examined whether turnover intention among social workers is shaped by the cumulative effects of past role conflict or by adaptive responses to changing work conditions. Contrary to H1, baseline RC did not significantly predict $TI_{\text{2}\text{0}\text{2}\text{2}}$ after controlling for $TI_{\text{2}\text{0}\text{1}\text{9}}$. However, H2 and H3 were supported, indicating that changes in RC and WS were significantly associated with changes in TI. The alternative baseline-adjusted specification yielded a consistent pattern, with RC and WS measured in 2022 remaining associated with TI in 2022 after their corresponding baseline levels and baseline TI were controlled. Taken together, the pattern of findings is more consistent with the adaptation perspective than with a persistent independent association of baseline RC with subsequent TI.

First, the results provide limited support for the accumulation perspective. Although baseline RC was positively associated with TI in 2022, this relationship became non-significant after controlling for TI measured in 2019. This finding indicates that baseline RC did not explain additional variation in subsequent TI beyond respondents’ pre-existing levels of TI [4,20,22,33]. In other words, the results do not provide evidence of a persistent independent association between earlier RC and subsequent TI once prior TI is taken into account [14,24]. The alternative baseline-adjusted specification produced a similar pattern: RC measured in 2019 was not independently associated with TI in 2022 when RC in 2022, baseline TI, WS at both waves, and demographic covariates were simultaneously considered.

Second, the results support the adaptation perspective. Changes in RC were significantly and positively associated with changes in TI. Social workers whose RC increased over time were more likely to report increases in TI, whereas reductions in RC were associated with decreases in TI. This finding is consistent with the adaptation perspective. One possible interpretation is that relatively stable work conditions may become incorporated into employees’ expectations over time, whereas deterioration or improvement in those conditions may prompt corresponding reassessments of whether to remain in the organization [8,18,24,30]. The present findings are consistent with this interpretation, although the two-wave design does not directly observe the underlying adaptation process.

Third, changes in WS were negatively associated with changes in TI. As a key job resource, WS may enhance employees’ ability to cope with work demands and maintain confidence in their work environment [17,18,34–36]. The findings indicate that increases in WS are associated with reductions in TI, whereas decreases in WS are associated with increases in TI. This result is consistent with the core proposition of the JD-R model that job resources help employees manage job demands and promote positive work outcomes [14,23,24]. The alternative baseline-adjusted specification provided convergent evidence: WS measured in 2022 remained negatively associated with TI in 2022 after WS in 2019, RC at both waves, and baseline TI were taken into account.

Overall, the convergence of findings across the lagged, change-score, and alternative baseline-adjusted specifications suggests that the observed longitudinal pattern is more consistent with an adaptation perspective than with a persistent independent association of baseline RC with subsequent TI. Baseline RC did not remain independently associated with later TI after baseline TI was considered, whereas within-person changes in RC and WS corresponded to changes in TI. These findings highlight the importance of considering changes in employees’ work conditions alongside their prior levels when examining TI longitudinally.

### Theoretical implications

This study contributes to the literature in two primary ways.

First, the study extends the application of the JD-R model by introducing a longitudinal perspective and distinguishing between accumulation and adaptation explanations of turnover intention. Existing research has largely assumed that the effects of job demands accumulate over time and continue to influence employees’ attitudes and behaviors [9,20,22,23,33,37]. However, the present findings suggest a different pattern. After controlling for prior turnover intention, baseline RC no longer significantly predicted subsequent TI, whereas changes in RC remained significantly associated with changes in TI. These findings indicate that turnover intention among social workers may be shaped less by the enduring effects of past role conflict and more by adaptive responses to changing work conditions. In doing so, this study provides longitudinal evidence that turnover intention is more strongly associated with changes in job demands than with the historical level of RC and enriches the explanatory scope of the JD-R framework.

Second, this study advances the conceptualization of RC as a job demand by emphasizing the importance of change rather than level. Previous studies have primarily examined RC as a relatively static stressor and focused on its direct effects on work outcomes [4,33,37]. In contrast, the present study suggests that changes in RC are more strongly associated with changes in TI than historical levels of RC. This finding suggests that employees appear to evaluate their work experiences in response to changes in working conditions and respond primarily to whether work conditions are improving or deteriorating [11,12,23]. By highlighting the importance of changes in work experiences over time, this study provides a new perspective for understanding how job demands influence employees’ career decisions over time.

The present study focuses on RC as a representative job demand and WS as a representative job resource. Although these two constructs capture key dimensions of the JD-R framework in the social work context, future research could incorporate additional job demands (e.g., workload and emotional demands) and job resources (e.g., autonomy and supervisory support) to develop a more comprehensive understanding of turnover intention.

## Conclusions

### Major findings

Grounded in the JD-R framework and based on matched panel data from the 2019 and 2022 waves of the CSWLS, this study examined the longitudinal relationships among RC, WS, and TI from the competing perspectives of accumulation and adaptation. By combining lagged outcome, change-score, and alternative baseline-adjusted analyses, the study provides evidence regarding how prior levels and changes in work conditions are associated with TI over time.

The findings provide limited support for the accumulation perspective. Although baseline RC was positively associated with subsequent TI in models without baseline TI, it was not independently associated with TI in 2022 once TI in 2019 was taken into account. Thus, the results do not provide evidence of a persistent independent association between baseline RC and subsequent TI beyond respondents’ prior levels of TI.

In contrast, changes in RC were positively associated with changes in TI, whereas changes in WS were negatively associated with changes in TI. The alternative baseline-adjusted specification yielded a consistent pattern: RC and WS measured in 2022 remained associated with TI in 2022 after their corresponding baseline levels and baseline TI were taken into account. Because the 2022 measures were assessed contemporaneously, these associations should not be interpreted as prospective or causal effects.

Overall, the convergence of findings across the different longitudinal specifications suggests that the observed pattern is more consistent with the adaptation perspective than with a persistent independent association of baseline RC with subsequent TI. Rather than demonstrating the underlying adaptation process directly, the findings highlight the importance of considering changes in employees’ work conditions alongside their prior levels when examining turnover intention longitudinally. By incorporating this temporal perspective into the JD-R framework, the study contributes to a more dynamic understanding of workforce stability among social workers.

### Limitations and future research

Despite its contributions, this study has several limitations that warrant consideration in future research.

First, although a longitudinal design was employed, the analysis was based on only two waves of panel data. Consequently, the findings should be interpreted as longitudinal evidence regarding changes in work conditions rather than as definitive evidence of dynamic processes. The study therefore captures changes over relatively broad intervals rather than continuous developmental trajectories. Future research using three or more waves of panel data and more advanced longitudinal modeling techniques would provide a stronger basis for examining adaptation and accumulation processes over time.

Second, although the present study applies the JD-R framework, it focuses on one representative job demand (RC) and one representative job resource (WS) to examine their longitudinal associations with TI. However, the JD-R framework encompasses a broader range of job demands and job resources. Future research could extend the present model by incorporating additional job demands (e.g., workload and emotional demands) and job resources (e.g., job autonomy and supervisory support) to provide a more comprehensive understanding of turnover intention among social workers.

Finally, this study focuses on social workers in China. Although the use of a nationwide dataset enhances the representativeness of the findings, institutional arrangements, organizational environments, and career structures differ across countries. Future research could adopt cross-national comparative designs to examine the generalizability of the findings across diverse institutional contexts and thereby strengthen external validity.

## Supporting information

S1 File This file contains S1–S7 Tables, including the sample construction process, variable construction, construction of change variables, attrition analysis, robustness analysis, codebook of study variables, and data processing and statistical analysis procedures. (DOCX)

## References

[1] Su X, Liang K, Wong V. The impact of collective psychological ownership on turnover intention among social service workers. Hum Serv Organ Manag Leadersh Gov. 2021;45:352–64. doi: 10.1080/23303131.2021.1898072

[2] Wang Y, Jiang N, Zhang H, Liu Z. Organizational justice, burnout, and turnover intention of social workers in China. Journal of Social Work. 2020;21(3):456–75. doi: 10.1177/1468017320911347

[3] Huo W, Shi B. Working overtime and turnover intention among Chinese social workers: roles of organizational identity and individual income. Sage Open. 2025;15(1). doi: 10.1177/21582440251323656

[4] Kim H, Stoner M. Burnout and turnover intention among social workers: effects of role stress, job autonomy and social support. Administration in Social Work. 2008;32(3):5–25. doi: 10.1080/03643100801922357

[5] Bi Y, Mou S, Wang G, Liao M. The relationship between professional self-concept and career decision-making difficulties among postgraduate nursing students in China: the mediating role of career decision-making self-efficacy. Front Psychol. 2023;14:1198974. doi: 10.3389/fpsyg.2023.1198974 37583606 PMC10424919

[6] Cellini G, Scavarda A. The effects of the economic crisis and managerialism in social work: A study of working practices and relationship reshaping. Alternativas Cuadernos de Trabajo Social. 2020;:9–25. doi: 10.14198/altern2020.27.01

[7] Li N, Peng J, Yang R. How do working conditions affect the turnover intention of medical social workers in China? BMC Health Serv Res. 2022;22(1):67. doi: 10.1186/s12913-021-07435-8 35031046 PMC8759249

[8] Tang X, Li X. Role stress, burnout, and workplace support among newly recruited social workers. Res Soc Work Pract. 2021;31(5):529–40. doi: 10.1177/1049731520984534

[9] Asayesh H, Haji Mohammad Hoseini M, Amaniyan S, Sharififard F, Elahi A, Yaghoubi Kopaie S. Role conflict and role ambiguity as predictors of turnover intention among nurses. J Nurs Midwifery Sci. 2021;8(1):49. doi: 10.4103/jnms.jnms_62_20

[10] Kerson TS, McCoyd JLM. In response to need: an analysis of social work roles over time. Soc Work. 2013;58(4):333–43. doi: 10.1093/sw/swt035 24450020

[11] Adomaitytė-Subačienė I. The role of social work knowledge in the creation of sustainable social innovation. Hum Serv Organ Manag Leadersh Gov. 2025;49:480–92. doi: 10.1080/23303131.2025.2554352

[12] Akabas SH, Gates LB. A social work role: promoting employment equity for people with serious and persistent mental illness. Adm Soc Work. 1999;23:163–84. doi: 10.1300/j147v23n03_10

[13] Browne T. Social Work Roles and Healthcare Settings. Handbook of Health Social Work. Wiley. 2019. 21–37. doi: 10.1002/9781119420743.ch2

[14] Demerouti E, Bakker AB, Nachreiner F, Schaufeli WB. The job demands-resources model of burnout. J Appl Psychol. 2001;86(3):499–512. doi: 10.1037/0021-9010.86.3.499 11419809

[15] Lesener T, Gusy B, Wolter C. The job demands-resources model: A meta-analytic review of longitudinal studies. Work & Stress. 2018;33(1):76–103. doi: 10.1080/02678373.2018.1529065

[16] Bakker AB, Demerouti E, Verbeke W. Using the job demands‐resources model to predict burnout and performance. Hum Resour Manag. 2004;43:83–104. doi: 10.1002/hrm.20004

[17] Li H, Mo J. The Relationship between Workplace Support and Turnover intention amongst Chinese Social Workers: A Moderated Mediation Model. Br J Soc Work. 2022;53(2):1055–76. doi: 10.1093/bjsw/bcac171

[18] Bakker AB, Demerouti E. The job demands‐resources model: state of the art. J Manag Psychol. 2007;22:309–28. doi: 10.1108/02683940710733115

[19] Jia CX, Li JC-M. Work-family conflict, burnout, and turnover intention among Chinese social workers: The moderating role of work support. J Soc Serv Res. 2022;48:12–27. doi: 10.1080/01488376.2021.1942393

[20] Ji J. Role conflict and turnover intention of Chinese social workers: a moderated mediation model. Asia Pac J Soc Work Dev. 2025;1–25. doi: 10.1080/29949769.2025.2498728

[21] Schaufeli WB, Bakker AB. Job demands, job resources, and their relationship with burnout and engagement: A multi‐sample study. J Organ Behav. 2004;25:293–315. doi: 10.1002/job.248

[22] Acker GM. The effect of organizational conditions (role conflict, role ambiguity, opportunities for professional development, and social support) on job satisfaction and intention to leave among social workers in mental health care. Commun Ment Health J. 2004;40(1):65–73. doi: 10.1023/b:comh.0000015218.12111.26 15077729

[23] Ployhart RE, Vandenberg RJ. Longitudinal research: The theory, design, and analysis of change. J Manag. 2009;36:94–120. doi: 10.1177/0149206309352110

[24] Demerouti E, Bakker AB. The Job Demands–Resources model: Challenges for future research. SA J Ind Psychol. 2011;37(2). doi: 10.4102/sajip.v37i2.974

[25] Han S-S, Han J-W, An Y-S, Lim S-H. Effects of role stress on nurses’ turnover intentions: The mediating effects of organizational commitment and burnout. Jpn J Nurs Sci. 2015;12(4):287–96. doi: 10.1111/jjns.12067 25469956

[26] Chen Y, Liang Z. Value compatibility: work–family conflict, psychological ownership, person–organisation value congruence and turnover intention amongst Chinese social workers. Br J Soc Work. 2023;54(4):1698–718. doi: 10.1093/bjsw/bcad223

[27] Schaufeli WB, Taris TW. A critical review of the job demands-resources model: implications for improving work and health. Bridging Occupational, Organizational and Public Health. Springer Netherlands. 2013. p. 43–68. doi: 10.1007/978-94-007-5640-3_4

[28] Ji J, Li Q, Qiu W, Li Y. Collective psychological ownership and turnover intention amongst social workers in China: the mediating effect of burnout. Asia Pac J Soc Work Dev. 2024;35(2):212–28. doi: 10.1080/29949769.2024.2384740

[29] Tabachnick BG, Fidell LS. Using multivariate statistics. 7th ed. New York, NY: Pearson. 2021.

[30] Rizzo JR, House RJ, Lirtzman SI. Role conflict and ambiguity in complex organizations. Adm Sci Q. 1970;15:150. doi: 10.2307/2391486

[31] Nissly JA, Barak MEM, Levin A. Stress, social support, and workers’ intentions to leave their jobs in public child welfare. Administration in Social Work. 2005;29(1):79–100. doi: 10.1300/j147v29n01_06

[32] Comer JM, Deeter-Schmelz DR, Ramsey RP. Methods in sales research: considering sources and types of social support: a psychometric evaluation of the house and wells (1978) instrument. J Pers Sell Sales Manag. 1997;17(1):49–61. doi: 10.1080/08853134.1997.10754082

[33] Soelton M, Lestari PA, Arief H, Putra RL. The Effect of Role Conflict and Burnout Toward Turnover Intention at Software Industries, Work Stress as Moderating Variables. In: Proceedings of the 4th International Conference on Management, Economics and Business (ICMEB 2019), 2020. 10.2991/aebmr.k.200205.034

[34] Gillman JC, Turner MJ, Slater MJ. The role of social support and social identification on challenge and threat cognitive appraisals, perceived stress, and life satisfaction in workplace employees. PLoS One. 2023;18(7):e0288563. doi: 10.1371/journal.pone.0288563 37437025 PMC10337949

[35] Khalil SM, Sher Nawaz K, Fayaz Ali S. The impact of work life balance, role conflict and work overload on employee turnover intention through the mediating role of job stress. J Bus Tour. 2020;6:271–87. doi: 10.34260/jbt.v6i1.194

[36] Cho Y, Choi S, Lee J. The study of the association between perceived value of work and turnover intention of social worker: analysis of the moderating effects of supervision. Cent Social Welf Res Yonsei Univ. 2019;61:45–78. doi: 10.17997/swry.61.1.2

[37] Asfahani AM. The impact of role conflict on turnover intention among faculty members: A moderated mediation model of emotional exhaustion and workplace relational conflict. Front Psychol. 2022;13:1087947. doi: 10.3389/fpsyg.2022.1087947 36619069 PMC9811317

