# Supplementary material for: Accumulation or adaptation? A longitudinal study of role conflict and turnover intention among Chinese social workers
Source: PLoS One. 2026 Oct 23;21(10):e0359307. doi: 10.1371/journal.pone.0359307 (PMC13693933; doi:10.1371/journal.pone.0359307)
Supplement: Supplementary file 1 [file pone.0359307.s001.docx]

**S1 File. Supporting Information**

Due to third-party restrictions imposed by the China Social Work Longitudinal Study (CSWLS) research team, the individual-level dataset used in this study cannot be publicly shared. To enhance transparency and reproducibility despite these restrictions, this Supporting Information provides detailed information on sample construction, variable operationalization, data processing, model specifications, and additional statistical analyses.

**S1 Table. Sample construction process.**

| **Stage** | **N** |
| --- | --- |
| **Valid respondents in 2019** | 5,965 |
| **Valid respondents in 2022** | 6,499 |
| **Successfully matched respondents** | 1,744 |

Note. The 1,744 successfully matched respondents constitute the longitudinal panel. Regression analyses used model-specific complete-case samples under listwise deletion; therefore, the effective sample size varied across regression specifications.

**S2 Table. Variable construction.**

| **Variable** | **Source** | **Items** | **Coding** | **Reliability** |
| --- | --- | --- | --- | --- |
| **RC** | Rizzo et al. [30] | 8 items | Mean score (1–5); higher scores indicate higher RC | **2019: α = 0.799; 2022: α = 0.831** |
| **TI** | Nissly et al. [31] | 3 items | Mean score (1–5); higher scores indicate stronger TI | **2019: α = 0.788; 2022: α = 0.790** |
| **WS** | Comer et al. [32] | 24 items | Mean score (1–5); higher scores indicate greater WS | **2019: α = 0.955; 2022: α = 0.960** |

Note. RC, role conflict; TI, turnover intention; WS, workplace support. All measures used five-point response scales, and higher mean scores indicate higher levels of the corresponding constructs. To ensure longitudinal comparability, the same items were used to construct each focal variable in both the 2019 and 2022 waves. RC was constructed using the eight items administered with equivalent wording across both waves. WS was constructed using the same 24 items in both waves, covering six aspects of workplace support across four organizational sources.

**S3 Table. Construction of change variables.**

| **Variable** | **Formula** |
| --- | --- |
| **ΔRC** | $\Delta RC=RC_{2022}-RC_{2019}$ |
| **ΔTI** | $\Delta TI=TI_{2022}-TI_{2019}$ |
| **ΔWS** | $\Delta WS=WS_{2022}-WS_{2019}$ |

Note. Positive values indicate increases between 2019 and 2022, whereas negative values indicate decreases over time.

**S4 Table. Attrition analysis.**

| **Variable** | **Attrited respondents** | **Retained respondents** | **p-value** | **Cohen’s d** |
| --- | --- | --- | --- | --- |
| **Age** | 33.22 | 33.90 | 0.002 | 0.085 |
| $\boldsymbol{R}\boldsymbol{C}_{\boldsymbol{2019}}$ | 2.75 | 2.76 | 0.611 | 0.015 |
| $\boldsymbol{T}\boldsymbol{I}_{\boldsymbol{2019}}$ | 2.61 | 2.43 | < 0.001 | 0.197 |
| $\boldsymbol{W}\boldsymbol{S}_{\boldsymbol{2019}}$ | 4.10 | 4.08 | 0.285 | 0.031 |

Note. Attrited respondents refer to individuals who participated in the 2019 survey but were not successfully matched in 2022. Retained respondents refer to individuals who participated in both survey waves. Valid sample sizes varied slightly across variables because of item-level missingness. According to Cohen’s (1988) criteria, all effect sizes were negligible to small in magnitude, suggesting limited evidence of substantial attrition bias.

**S5 Table. Robustness analysis.**

| **Variable** | **B (SE)** |
| --- | --- |
| **Gender** | −0.042 (0.048) |
| **Age** | −0.016^***^ (0.003) |
| **Marital status** | −0.077 (0.044) |
| **Party membership** | −0.028 (0.046) |
| **Education** | −0.012 (0.015) |
| **Income** | −0.021^*^ (0.009) |
| **RC_2019_** | −0.058 (0.035) |
| **RC_2022_** | 0.234^***^ (0.032) |
| **WS_2019_** | 0.018 (0.029) |
| **WS_2022_** | −0.225^***^ (0.027) |
| **TI_2019_** | 0.223^***^ (0.024) |
| **Observations (N)** | 1,612 |
| **F-statistics** | 44.328^***^ |
| **R²** | 0.234 |
| **Adjusted R²** | 0.228 |

Note. Unstandardized coefficients (B) are reported with standard errors in parentheses. The model regresses TI2022 on RC2019, RC2022, WS2019, WS2022, and TI2019, while controlling for gender, age, marital status, party membership, education, and income. * p < 0.05, ** p < 0.01, *** p < 0.001.

**S6 Table. Codebook of study variables.**

| **Variable** | **Description** | **Operationalization** |
| --- | --- | --- |
| **Gender** | Respondent's gender | 0 = Male; 1 = Female |
| **Age** | Respondent's age | Years |
| **Marital status** | Marital status | 0 = Unmarried; 1 = Married |
| **Party membership** | CPC membership | 0 = Non-CPC member; 1 = CPC member |
| **Education** | Educational attainment | Years of education |
| **Income** | Monthly income | Ordinal scale provided in CSWLS |
| $\text{R}\text{C}_{\text{2019}}$ | Role conflict (2019) | Mean score of 8 items |
| $\text{R}\text{C}_{\text{2022}}$ | Role conflict (2022) | Mean score of 8 items |
| $\text{T}\text{I}_{\text{2019}}$ | Turnover intention (2019) | Mean score of 3 items |
| $\text{T}\text{I}_{\text{2022}}$ | Turnover intention (2022) | Mean score of 3 items |
| $\text{W}\text{S}_{\text{2019}}$ | Workplace support (2019) | Mean score of 24 items |
| $\text{W}\text{S}_{\text{2022}}$ | Workplace support (2022) | Mean score of 24 items |
| **ΔRC** | Change in role conflict | RC2022 − RC2019 |
| **ΔTI** | Change in turnover intention | TI2022 − TI2019 |
| **ΔWS** | Change in workplace support | WS2022 − WS2019 |

Note. RC, role conflict; TI, turnover intention; WS, workplace support. Composite variables were calculated as the mean of their corresponding items. To ensure longitudinal comparability, the same items were used to construct each focal variable in both survey waves. Change variables were calculated as the 2022 score minus the corresponding 2019 score, such that positive values indicate increases and negative values indicate decreases over time.

**S7 Table. Data processing and statistical analysis procedures.**

| **Step** | **Procedure** |
| --- | --- |
| **1** | Import the 2019 and 2022 CSWLS datasets. |
| **2** | Match respondents across the two survey waves using unique respondent identification codes to construct the longitudinal panel. |
| **3** | Recode user-defined missing values (e.g., −9999) as system-missing values. |
| **4** | Compute composite scores for RC, TI, and WS by averaging the corresponding questionnaire items, using the same items across the two waves to ensure longitudinal comparability. |
| **5** | Construct change variables (ΔRC, ΔTI, and ΔWS) by subtracting the corresponding 2019 scores from the 2022 scores. |
| **6** | Conduct descriptive statistics and Pearson correlation analyses. |
| **7** | Assess potential common method bias and multicollinearity using Harman’s single-factor test and variance inflation factors (VIFs), respectively. |
| **8** | Estimate the lagged outcome models and change-score models, with missing observations handled by listwise deletion on a model-specific basis. |
| **9** | Estimate an alternative baseline-adjusted specification including RC and WS measured at both waves, baseline TI, and demographic covariates as a robustness analysis. |

Note. RC, role conflict; TI, turnover intention; WS, workplace support. All statistical analyses were performed using SPSS Statistics Version 22.0. The matched longitudinal panel comprised 1,744 respondents. Regression analyses used listwise deletion, resulting in model-specific analytical sample sizes. No missing values were imputed. Detailed model specifications are provided in the Methods section of the manuscript.
